# OPTIMIZING ONLINE DELIVERY OF INTERGENERATIONAL STUDENT EXPERIENCES: LEVERAGING OPPORTUNITIES

**DOI:** 10.1093/geroni/igac059.1052

**Published:** 2022-12-20

**Authors:** Lisa Borrero

**Affiliations:** University of Indianapolis, Indianapolis, Indiana, United States

## Abstract

Conceptualizing intergenerational opportunities for students in fully online courses can initially appear daunting or impractical, given the absence of a common physical classroom and community environment. However, it could be argued that online learning serves as a facilitator, rather than a barrier to intergenerational programming. This session will focus on the connections between intergenerational programming and the principles of quality online teaching – emphasizing the ability of online modalities to increase connections with a global community, remove the limitations of geographic-based access to experiences, and elevate the level of student engagement typically expected within online courses. Leveraging opportunities to overcome real and perceived challenges to the planning, delivery, and evaluation of intergenerational student experiences in online courses will be discussed, as well as opportunities for future growth in the current era.

